# The use of Unmanned Aerial Vehicles (UAVs) to sample the blow microbiome of small cetaceans

**DOI:** 10.1371/journal.pone.0235537

**Published:** 2020-07-02

**Authors:** Cinzia Centelleghe, Lisa Carraro, Joan Gonzalvo, Massimiliano Rosso, Erika Esposti, Claudia Gili, Marco Bonato, Davide Pedrotti, Barbara Cardazzo, Michele Povinelli, Sandro Mazzariol

**Affiliations:** 1 Department of Comparative Biomedicine and Food Science, University of Padua, Padua, Italy; 2 Tethys Research Institute, Milan, Italy; 3 CIMA Research Foundation, Savona, Italy; 4 Costa Edutainment spa c/o Acquario di Genova, Genova, Italy; 5 Stazione Zoologica Anton Dohrn, Naples, Italy; 6 Department of Biology, University of Padua, Padua, Italy; Universidad de los Andes, COLOMBIA

## Abstract

Recent studies describe the use of UAVs in collecting blow samples from large whales to analyze the microbial and viral community in exhaled air. Unfortunately, attempts to collect blow from small cetaceans have not been successful due to their swimming and diving behavior. In order to overcome these limitations, in this study we investigated the application of a specific sampling tool attached to a UAV to analyze the blow from small cetaceans and their respiratory microbiome. Preliminary trials to set up the sampling tool were conducted on a group of 6 bottlenose dolphins (*Tursiops truncatus*) under human care, housed at Acquario di Genova, with approximately 1 meter distance between the blowing animal and the tool to obtain suitable samples. The same sampling kit, suspended via a 2 meter rope assembled on a waterproof UAV, flying 3 meters above the animals, was used to sample the blows of 5 wild bottlenose dolphins in the Gulf of Ambracia (Greece) and a sperm whale (*Physeter macrocephalus*) in the southern Tyrrhenian Sea (Italy), to investigate whether this experimental assembly also works for large whale sampling. In order to distinguish between blow-associated microbes and seawater microbes, we pooled 5 seawater samples from the same area where blow samples’ collection were carried out. The the respiratory microbiota was assessed by using the V3-V4 region of the 16S rRNA gene via Illumina Amplicon Sequencing. The pooled water samples contained more bacterial taxa than the blow samples of both wild animals and the sequenced dolphin maintained under human care. The composition of the bacterial community differed between the water samples and between the blow samples of wild cetaceans and that under human care, but these differences may have been mediated by different microbial communities between seawater and aquarium water. The sperm whale’s respiratory microbiome was more similar to the results obtained from wild bottlenose dolphins. Although the number of samples used in this study was limited and sampling and analyses were impaired by several limitations, the results are rather encouraging, as shown by the evident microbial differences between seawater and blow samples, confirmed also by the meta-analysis carried out comparing our results with those obtained in previous studies. Collecting exhaled air from small cetaceans using drones is a challenging process, both logistically and technically. The success in obtaining samples from small cetacean blow in this study in comparison to previous studies is likely due to the distance the sampling kit is suspended from the drone, which reduced the likelihood that the turbulence of the drone propeller interfered with successfully sampling blow, suggested as a factor leading to poor success in previous studies.

## Introduction

The use of Unmanned Aerial Vehicles (UAVs or drones) has been increasingly successfully applied to wildlife monitoring for conservation activities over the past decade [1]. UAVs have also been implemented in research on marine megafauna, including cetaceans, mainly to investigate abundance, distribution, habitat, and measurement of the individuals through photogrammetry methods, offering an opportunity to decrease costs, noise, and carbon emissions, improve precision of counts, accuracy and resolution of sighting location data, and allow surveys to be conducted within a larger range of environmental conditions than traditional aerial surveys [2,3,4,5,6,7,8]. In 2010, Acevedo-Whitehouse described the possibility of collecting cetacean blow samples using UAVs to analyze respiratory bacteria [9]. More recently, Apprill and colleagues (2017) described the use of UAVs for collecting blow samples from Eastern Australian humpback whales **(***Megaptera novaeangliae*) to analyze the large core microbiome in the exhaled air [10], while Geoghegan and colleagues (2018) were able to investigate virome of the same cetacean species by using drones [11]. Cetacean blow samples collected using UAVs could also be used to evaluate hormonal levels and contribute to the physiological assessment of large whales [12]. Moreover, the use of aerial vehicles was proposed by Raverty and colleagues (2017) to foster the analysis of exhaled breath as a minimally invasive method to evaluate respiratory microbiome, metabolites and hormones by coupling sampling with visual assessment [13].

While the use of UAVs for blow sampling in wild whales is increasingly applied due to the slow swimming speed of whales and diving patterns that facilitate relatively easy sampling, and Frere and colleagues (2010) reported the possibility of collecting dolphin DNA from the blow of animals under human care [14], this technique has unsuccessfully been applied for blow sampling in wild dolphins, mainly because of their fast and unpredictable behavior [15], although UAVs have been applied to ecological and behavioral research [2]. Conservation objectives included in relevant EU Directives (e.g. Marine Strategy Framework Directive) have highlighted the need to increase the knowledge on the biology and ecology of small odontocetes. Therefore, here we investigate the use of a sampling tool suspended below a UAV to overcome the previous limitations of using UAVs to collect blow samples for respiratory microbiome analyses in small cetaceans.

## Materials and methods

### Sample collection

**Fig 1:**
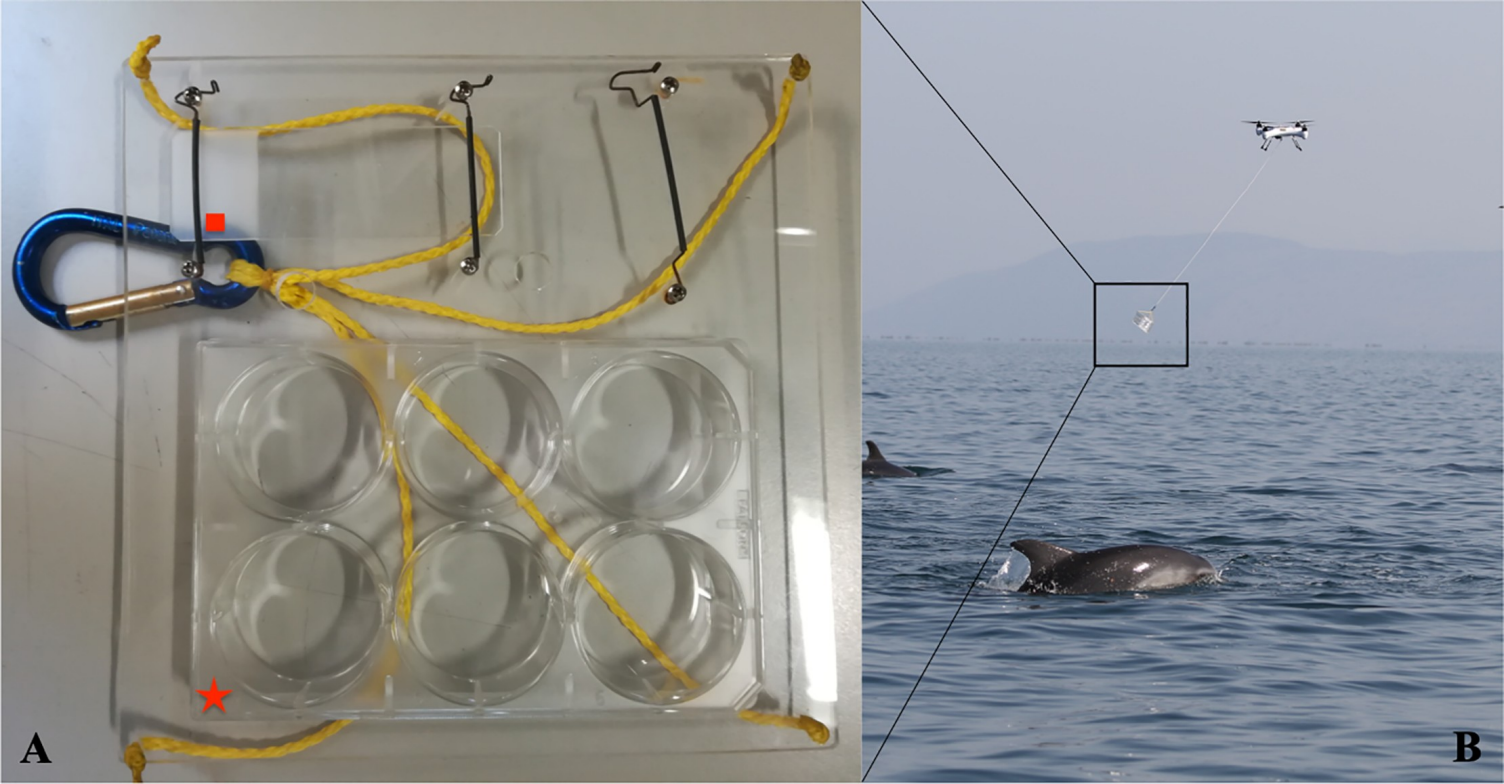
Sampling tool. (A) The tool composed by a 6-well polystyrene sterile Petri plate (Falcon^TM^), marked with a red star, and a cytology glass, marked with a red square, mounted on a Plexiglas support. (B) The unmanned quadcopter drone (SplashDrone, Swellpro Technology Co., Ltd) is used to collect blow samples via the sampling tool suspended at 2 m from the drone shell.

Our novel sampling tool (Fig 1A) consisted of a 6-well polystyrene sterile Petri plate (Falcon^TM^) and a cytology glass embedded in a Plexiglas support, suspended 2 meters (m) below the drone shell (Fig 1B). The tool was optimized after conducting preliminary trials on 6 bottlenose dolphins (*Tursiops truncatus*, hereafter referred to as bottlenose dolphin) maintained under human care at Acquario di Genova and, at the same time, the optimal sampling height above the animal for gathering appropriate bacterial samples was established. Furthermore, even if it was not the main goal of this study, any possible behavioral disturbances caused by the UAVs’ noise and presence were collected in wild animals by experienced marine biologists to report any stressful condition or changes in the behavior likely related to the flying object.

**Fig 2:**
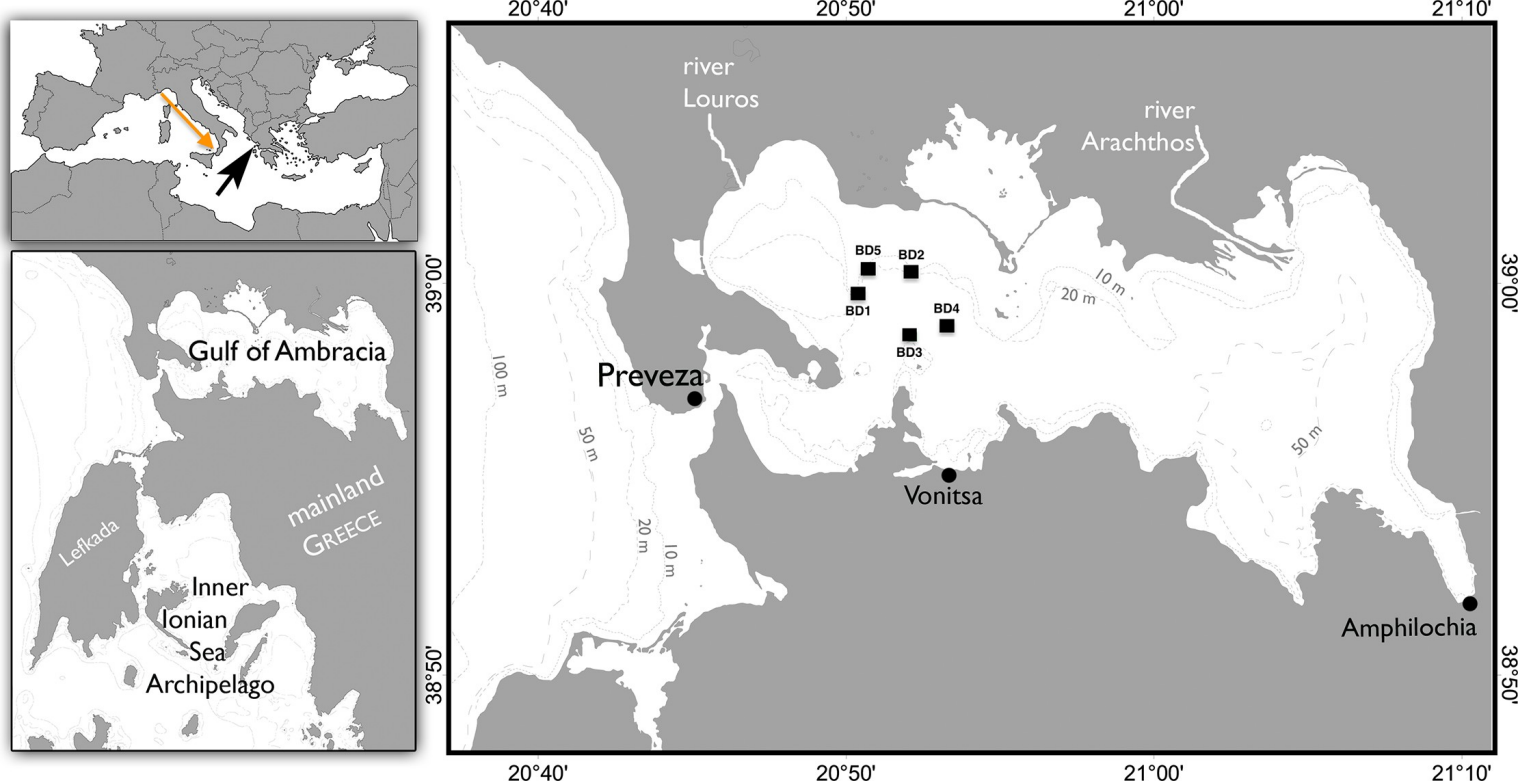
Maps of the Gulf of Ambracia. Geographic origin of wild bottlenose dolphins and sperm whale sampled during the study. Orange arrow: sampling site for the sperm whale in the Thyrrhenian Sea. Black arrow: geographical localization of The Gulf of Ambracia. The Gulf is a shallow, semi-closed embayment of 405 km^2^ whose only communication with the open Ionian Sea is through the Preveza Channel, a narrow (minimum width of 370m) and shallow (2–12 m deep) 3 km-long corridor. Right map: Blows correspond to Table 1 IDs.

Samples of exhaled breath condensate (blows) were collected from 5 wild bottlenose dolphins living in the Gulf of Ambracia (northwestern Greece) in July 2017 (Fig 2 for the exact locations), 1 animal maintained *ex-situ* in an Italian aquarium (Acquario di Genova) and 1 sperm whale (*Physeter macrocephalus*) in the southern Tyrrhenian Sea (Lat 38.47°N; Long 15.61°E) in August 2017 (Table 1).

**Table 1:** Data of sampled animals.

ID	Species	Sampling location	Date of sampling	Wild or Under human care (UHC)
BD1	Bottlenose dolphin	Gulf of Ambracia, Lat 38.99°N; Long 20.51°E	25.07.2017	Wild
BD2	Bottlenose dolphin	Gulf of Ambracia, Lat 39.01°N; Long 20.61°E	26.07.2017	Wild
BD3	Bottlenose dolphin	Gulf of Ambracia, Lat 38.93°N; Long 20.61°E	27.07.2017	Wild
BD4	Bottlenose dolphin	Gulf of Ambracia, Lat 38.94°N; Long 20.66°E	27.07.2017	Wild
BD5	Bottlenose dolphin	Gulf of Ambracia, Lat 39.01°N; Long 20.53°E	27.07.2017	Wild
SW6	Sperm whale	Tyrrhenian Sea, Lat 38.47°N; Long 15.61°E	09.08.2017	Wild
BDc7	Bottlenose dolphin	Genova Aquarium, Genova (Italy)	18.09.2016	UHC

Samples from 5 wild bottlenose dolphins were collected on 3 different days using a waterproof four-bladed helicopter (diagonal diameter of 550 mm, 2.3 kg, carbon fiber propellers, Splash Drone, Swellpro Technology Co. Ltd, Shenzhen, China) operated by a pilot and copilot team from a small vessel. Animals were engaged during feeding activities or in traveling mode and the drone was flown over them only for the duration of the sampling (from 3 to 5 minutes). Sample sites were recorded and seawater samples were taken on the same day and site of blow sampling, in order to distinguish between blow-associated microbes and seawater microbes. 10 ml of seawater was collected from the surface down to 0.25 m depth using a 15 ml sterile tube mounted on a previously sterilized plastic pole. No seawater samples were collected during the sampling of the dolphin kept under human care since we used this animal just to set our protocols as preliminary study on the sampling tool; also, in the case of the sperm whale no water samples were collected because it was not a planned activity.

Despite bleach and UV being the gold standard to destroy any residual of microbial nucleic acid, due to field conditions, prior to flight, the propellers, arms, struts, and dome were sterilized with 95% ethanol. During sampling, face masks, gloves, and sterile plastic material (Petri plate and pipette filter tips) were used to collect each blow sample, changing Petri plates after each sampling effort, to reduce any possible contamination. Then, the drone flew at around 3 m above the blowhole (as visually assessed via binoculars from the boat), and once the dolphin exhaled, releasing a poorly visible cloud of water vapor, the quadcopter returned to the boat where the sample was processed immediately. In order to avoid any potential contamination when animals were grouped, one flight over any single dolphin was performed. Next, each Petri dish 6-well that contained droplets from the blow was rinsed carefully with 1,5 ml of sterile RNA-later solution (Thermo Fisher Scientific) and the solution was collected in a sterile 2 ml cryovial, and frozen at -80°C until further processing.

Cytology specimens were air-dried and then stained using the May-Grunwald-Giemsa technique [16].

The dolphins maintained under human care mentioned in this work were handled and maintained in an artificial environment at Acquario di Genova (Ponte Spinola, 16128 Genova Italy). This is an aquarium facility approved by Italian Zoo law (Dlgs 73/2005 that derives from the European Zoo Directive 22/1999). All the samples obtained (*in vivo* diagnostic blow samples) were not invasive and collected during routine veterinary examinations, performed according to the Italian D.M. 469/2001 and to the law mentioned above, which establishes the management objectives and prescriptions to maintain the species bottlenose dolphins under human care in Italy.

Regarding the sampling in wild bottlenose dolphin living in the Gulf of Ambracia (Greece), the Tethys Research Institute has obtained permit no 147434/2810 for the period (2017–2019) to perform studies on live wild cetaceans and to collect samples from specimens with permission of the Hellenic Ministry of Environment and Energy.

Concerning the sapling in the wild sperm whale in the Southern Tyrrhenian Sea, the CIMA Research Foundation has the permit no MATTM PNM II 0012271 to perform studies on live wild cetaceans and to collect samples from specimens with permission of the Italian Ministry for Environment, Land and Sea Protection.

### Sample preparation, PCR amplification and sequencing

Nucleic acids were isolated from each (5 wild, 1 dolphin under human care and 1 sperm whale) of the 1.5 ml RNA-later solution using a commercial kit (DNeasy Blood & Tissue Kit, QIAGEN) according to the manufacturer protocol “Purification of Total DNA from Animal Blood or Cells (Spin-Column)” and the “Pretreatment protocol for Bacteria” to lyse bacterial cell walls before DNA purification. The 5 seawater samples from the Gulf of Ambracia were singularly centrifuged, and the resulting pellet was used for DNA extraction. The DNA extracted from the different seawater samples was subsequently pooled and microbiome analyses were performed on the single pool of DNA samples.

The V3-V4 region of the 16S rRNA bacterial and archaeal gene was amplified using the 341F and 806R primers [17]. PCR were carried out in 20 μL volumes per sample (plus 5 μL of purified sample DNA) containing 13,8 μL H_2_O, 2,5 μL 5X Phusion HF Buffer, 1 μL 10mM dNTPs, 0.5 μL 50 mM MgCl_2_, 1 μL 10mM forward primer and 1 μL 10mM reverse primer, and 0,2 μL Phusion Hot Start II High-Fidelity DNA Polymerase (2 U/μL) (Phusion Hot Start II DNA Polymerase, Thermo Fisher Scientific). Thermal cycling conditions using Bio-Rad Thermocycler (Hercules, CA) were an initial denaturation step at 94°C for 1 min; 30 cycles for seawater and 35 for blow samples of 94°C for 30 s, 55°C for 30 s, and 68°C for 45 sec; and an extension step at 68°C for 8 min.

Products of the PCR reaction were screened on a 2% agarose–Tris-acetate-EDTA (TAE) gel using HyperLadder (50bp; Bioline USE Inc., Taunton, MA) to confirm amplicon size. Negative controls both in DNA extraction and PCR steps were included and they did not result in any visible bands when run on agarose gels.

25 μl of PCR products from the 5 wild dolphins, the sperm whale, 1 dolphin kept under human care and the pooled seawater were submitted for library preparation to BMR Genomics (Padova, Italy). Barcodes were added by a second step of amplification and final libraries were sequenced using the Illumina MiSeq platform with a paired-end 300-cycle run.

### Sequence data processing and microbial community analysis

Raw sequences (356514, with an average of 59400 reads per sample) were processed using the QIIME2 software (2017.11). Data files were imported into a QIIME2 artefact using the 'SampleData[PairedEndSequencesWithQuality]' semantic type. Taxa that have less than 0.01 percent relative abundance across all samples have been removed from the analysis [18]. For sequence quality control and feature table construction (Amplicon Sequence Variant, ASV, table), the DADA2 pipeline was employed [19]. Forward and reverse reads were quality filtered, truncated at 260 bp and merged together. To assign taxonomy to the sequences, the Naive Bayes classifier and the q2-feature-classifier plugin were used [20] and Naive Bayes sklearn-based taxonomy classifier as pre-fitted on the Green genes 13_8 was employed. The raw sequence data were deposited in the SRA database with accession number PRJNA509077. The statistical analysis was performed using Calypso software (http://cgenome.net/calypso/) [21].

### Meta-analysis of microbiomes from cetacean respiratory tract and water samples

Since our study was strongly limited by field conditions in sampling and analyses, in order to increase the confidence of the obtained data, we decided to perform a meta-analysis comparing our results with published sequences obtained from cetaceans’ exhaled air samples and the relative sea water control samples. Considering that the use of different sequencing platforms can affect the characterization of microbiomes [22] and that different 16S regions have different resolutions [23], only data reported by Apprill and colleagues [10] were used for this study: microbiome sequences from 41 humpback whale and 18 seawater samples obtained by Miseq Illumina sequencing and targeting the 16S rRNA V3 region [10] were incorporated with our dataset. Raw sequence data (PRJNA401637) was downloaded from NCBI server (https://www.ncbi.nlm.nih.gov/sra), splitted and coverted by using the SRA toolkit (https://github.com/ncbi/sra-tools/wiki/01.-Downloading-SRA-Toolkit). Sequences were imported in QIIME2 and processed with the same pipeline described in the previous paragraph. The feature-classifier extract-reads plugin was employed to extract V3 region from our data. ASV tables were then collapsed togheter. Final table and taxonomy files were uploaded in Calypso and a 3D PCoA plot was used to evaluate the beta-diversity between the 67 samples.

## Results

Preliminary studies conducted on dolphins kept under human care suggested that the 6-well sterile Petri plate for blow collection should be placed at a maximum of 1 m above the blowhole, ensuring both a sufficient amount of microbial DNA for NGS studies and the presence of the sample on the cytological glass. It was decided to develop the sampling tool with the plate suspended by a 2 m-long rope maintained at 1 m from the blowhole, to avoid possible disturbance to wild dolphins during sampling procedures using UAV. Despite this study not being aimed at assessing any behavioral changes related to the use of flying drones on wild dolphins, no effect on behavior was noted during sampling activities likely caused by the close distance of UAVs.

**Fig 3:**
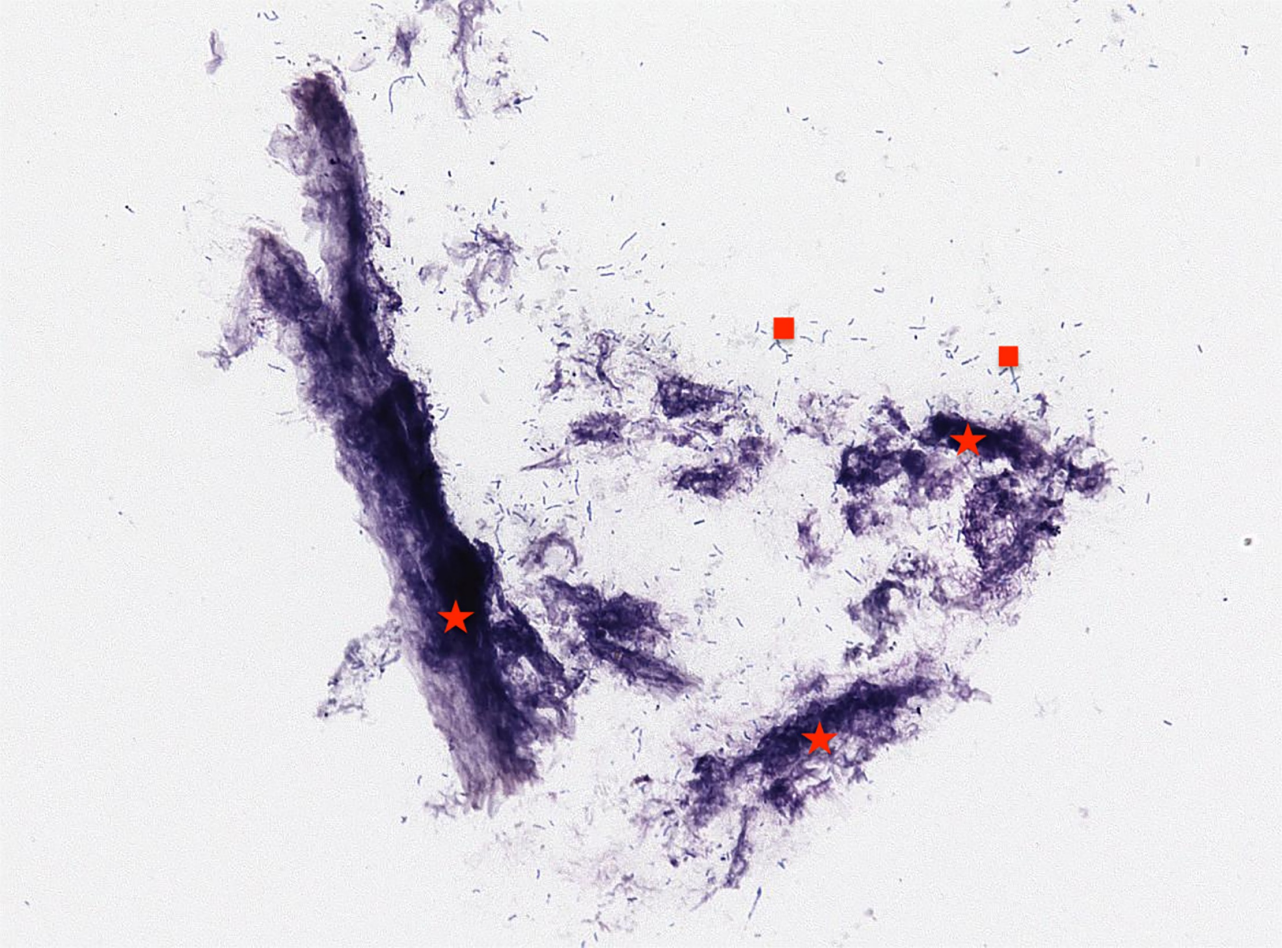
Cytological smears. Different bacterial elements, marked with red squares, along with sloughed keratinized epithelial scales, marked with red stars (May-Grunwald Geimsa staining, 40X).

Microscopic examination of cytological smears confirmed sampling success: different bacterial elements along with sloughed keratinized epithelial scales were observed (Fig 3).

**Fig 4:**
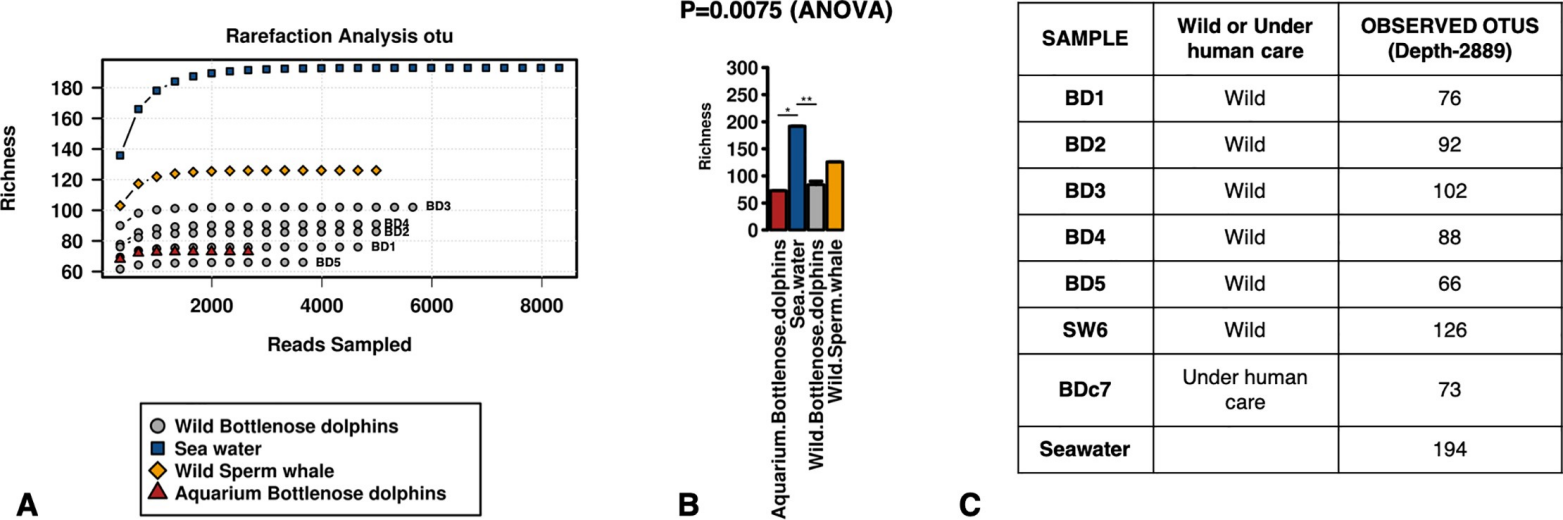
Alpha-diversity. Community richness evaluation by rarefaction analysis (A) and by ANOVA comparison of samples from different habitat (B), and the individual values of the observed OTUS (C).

**Fig 5:**
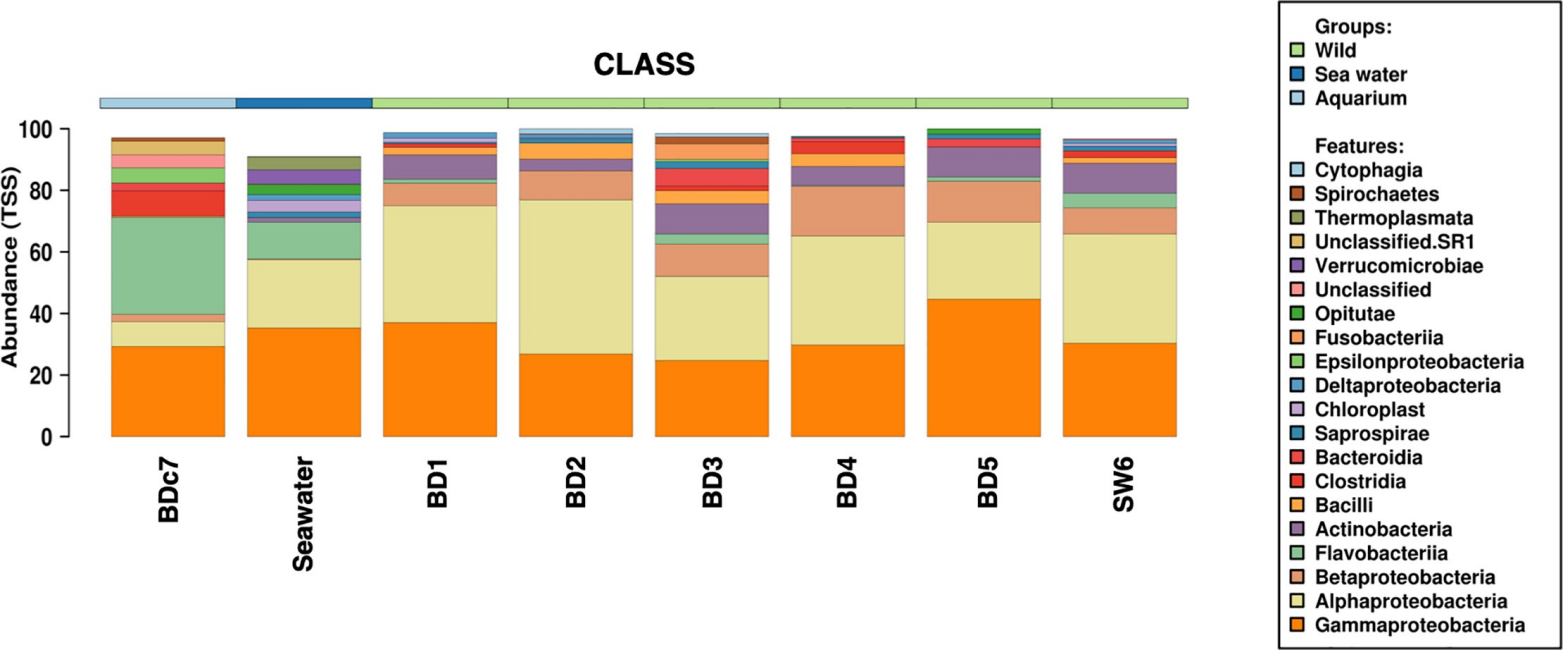
Bacterial community composition. Bacterial community composition at Class level in blow from wild bottlenose dolphins, the one under human care bottlenose dolphin, sperm whale and seawater.

The number of the reads obtained after sequencing for each sample was enough to describe the microbial community profiles, as indicated by the rarefaction curve slope (Fig 4). These communities differed in the alpha-diversity *richness* and *chao1* (not shown) indices. As expected, the water samples contained more bacterial taxa (p = 0.022), while the samples from wild animals (bottlenose dolphins and the sperm whale) and the aquarium animal were similar in taxa richness (Fig 4B). The bacterial communities were composed primarily of *Proteobacteria* (*Alphaproteobacteria* and *Gammaproteobacteria*) followed by *Bacteriodetes*, *Actinobacteria* and *Firmicutes*. The main classes composing the bacterial communities are presented in Fig 5.

**Fig 6:**
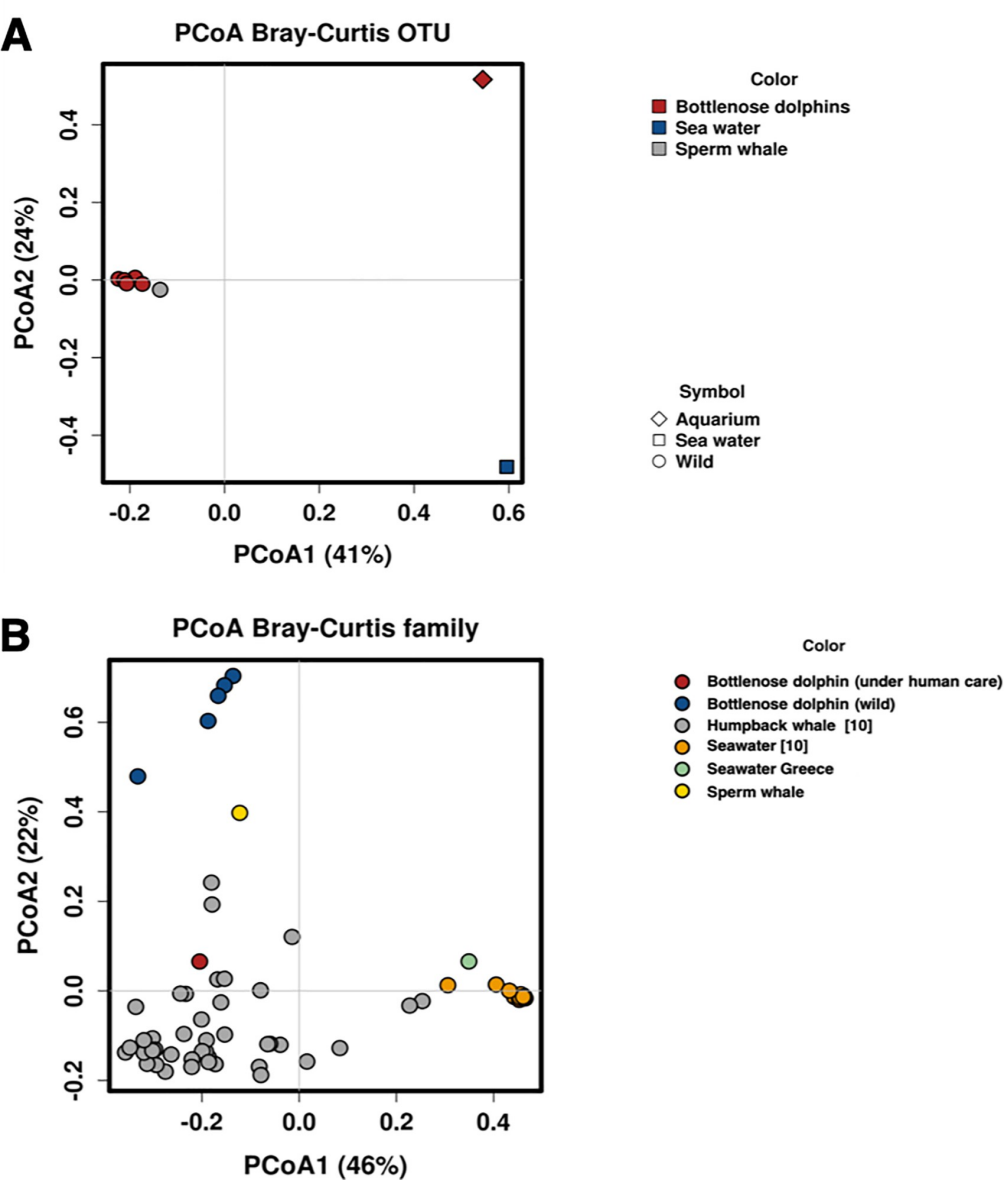
Principal coordinate analysis (PCoA): (A) PCoA of blow microbial community from wild and under human care bottlenose dolphins, sperm whale, and the pooled seawater sample. It’s possible to visualize a greater similarity between different species when sampled in the wild (sperm whale and bottlenose dolphin samples), while there was distance between the same species (bottlenose dolpdhin) sampled in wild vs. kept under human care. Seawater communities were distinct from all other samples. (B) PCoA of blow microbial community obtaining from meta-analysis by adding public data [10] to our dataset.

The composition of the bacterial communities was compared using Principal Coordinate Analysis (PCoA) among samples from wild animals (bottlenose dolphins in Greece and sperm whale in the Tyrrhenian Sea), seawater and the under human care dolphin. Due to field limitations, this analysis also included control and sequences already reported by Apprill and colleagues in *Megaptera novaeangliae* [10] to compensate the lack of consistent controls from the sea water and the aquarium water sampled in this study. The results of the PCoA (Fig 6) confirmed the distance between the microbial community of seawater and blows, from bottlenose dolphins and humpback whales and a difference between blows of wild animals with respect to the one kept in aquarium.

**Fig 7:**
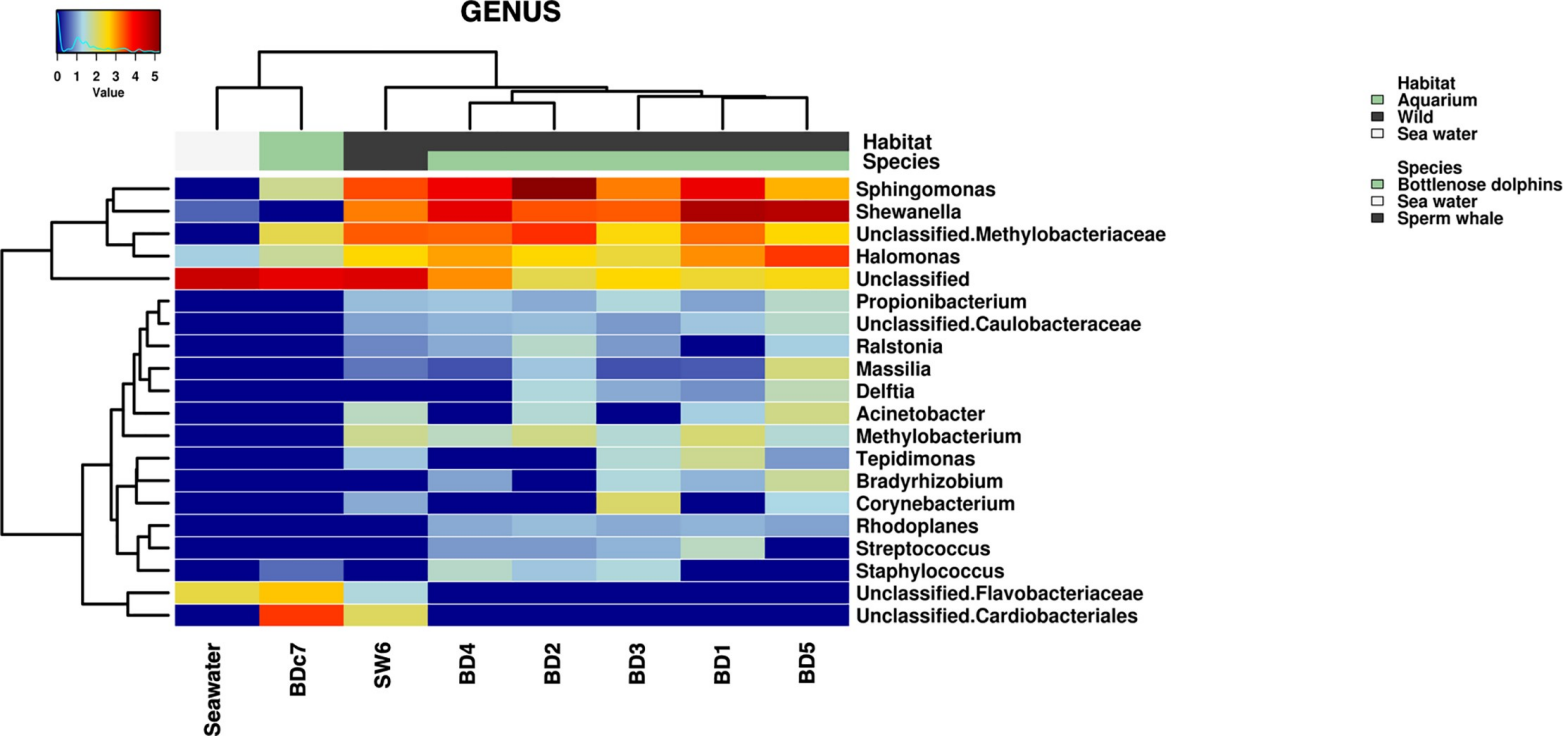
Hierarchical clustering of blow and the pooled water sample based on the most aboundant Genera by looking at the top 20 taxa in each sample. Taxa abundance are presented in color code ranging from red (highly abundant) to blue (rare or absent).

In term of *Genera* composition, the blow samples shared predominant bacterial genera such as *Sphingomonas*, *Shewanella*, *Halomonas* and an unclassified genus of Methylobacteriaceae, most of which were absent in the seawater samples (Fig 7). It should be noted that due to field conditions, the analyzed water samples were a pool from 5 different locations and we did not include a water control from the Aquarium, which limit the comparison.

## Discussion

Collecting blow samples using UAVs represents an interesting non-invasive, but challenging technique in obtaining information on the health status of small cetaceans. Swimming speed, unpredictable swimming pattern, respiratory rate, and social structure of small dolphin species complicate the adoption of the UAV’s sampling approach typically used in large whales [5, 10, 15]. Furthermore, the necessity for relatively close approaches above the dolphins and the possible noise produced by UAV rotors could enhance a disturbance through curiosity, avoidance and stress response in the dolphins [24, 25]. Although observing behavioral changes was not the main of our study, during the sampling procedure using UAV, no evident changes in dolphin swimming patterns or anomalous behavior caused by the presence of the drone were noted, possibly related to the distance of the flying vehicle. Previous studies using the same drone reported redirection and behavioral changes related to stress due to the presence of UAVs flying at a height of 10 m for several minutes, while the animals were resting [25]. Possible differences were potentially related to the distance of the drone from the sea surface and to the fact that sampling occurred for a short period of time when animals were feeding or traveling: the length of the duration, the distance of the flying vehicles and the activities of the animal were the reason of no evident avoidance response.

The sampling tool used in this study was developed and the methodology adapted to bottlenose dolphins maintained in an aquarium, considering both sampling success and safe distance from the animals, that did not compromise their normal behavior. Our results, despite being acquired from a small number of animals, suggest that this approach could represent a first step in overcoming the difficulties, already reported in other studies, associated with a drone-based sampling technique for wild small cetaceans [15]. A major limitation with using UAVs for blow sampling is the impossibility to match the samples with the individuals because bottlenose dolphin groups are relatively large, densely concentrated and highly dynamic, with frequent changes in group size and composition of individuals [26]. The contemporary use of a sampling tool with a camera mounted on the UAV, as already tested by Raudino and colleagues (2019) [15], was not possible in our study; this technical limitation should be addressed in future studies by using different and more proficient UAVs. Another potential method to link blow samples to individual animals could be searching for the host DNA in the blow sample, which could provide a genetic individual identification.

Before discussing the real effectiveness of this methodology and the results obtained, we have to stress again that the study herein presented was strongly affected by the main goal of the study (tuning of a drone based equipment for blow collection in small cetaceans) and by difficult field conditions (i.e. distance from a laboratory supporting sterilizing procedures with UV, sample storage or delivery) which impaired the sampling efforts (impossibility to follow standard procedures, lack of controls) and represent a caveat for the subsequent analyses and evaluation.

Despite these limitations, the effectiveness of sampling efforts under field conditions was confirmed by the cytological smears and by the variety in the microbial community characterizing the blows of wild dolphins and the differences between blow samples and seawater microbiota, similar to those previously described in large whales [5, 10]. In particular, the meta-analyses carried out comparing our results with sequences obtained in humpback whales from Apprill and colleagues [10] support the consistency of our data, with obvious differences due to the species considered. The pooled water sample presents a statistically significant larger microbial biodiversity compared to the bacterial phyla detected in exhaled air, as reported in the ANOVA comparison of samples from different matrixes. Furthermore, the microbiota is similar amongst all 5 bottlenose dolphins sampled at sea, while this composition differs from samples obtained from the sperm whale and the dolphin held in the aquarium. It should be noted that, even given results obtained from the latter are quite distinct from the sea water, the lack of aquarium water controls means we cannot rule out the distinctiveness could be due to background differences in the microbial communities between seawater and aquaria. Regarding the bacterial Genera, some of them, namely, *Sphingomonas sp*., *Streptococcus sp*. and *Staphylococcus spp*., were found both in wild and aquarium bottlenose dolphins, while *Acinetobacter sp*. and *Corynebacterium sp*. were found only in wild cetaceans [27,28,29,30]. Same of these Genera were already been reported in blow of humpback whales [10] and killer whales [13]. Obviously this kind of analysis cannot assess their potential pathogenic role in causing respiratory diseases: some of them are routinely found in mammals respiratory tract microbiome (i.e *Staphylococcus*, *Streptococcus*, *Propionibacterium* and *Corynebacterium*) with *Propionibacterium* reported only in cetaceans [31]. Furthermore, *Staphylococcus*, *Streptococcus*, and *Corynebacterium* along with *Shewanella* have been recently reported as a constant component of the external skin microbiota close to the blowhole external skin [28, 32]. Since *Staphylococcus*, *Streptococcus* and *Corynebacterium* have also been reported in the healthy human respiratory microbiota [33, 34], we could not exclude any possible field contamination which may have altered results despite our use of sterile gloves and face masks during sampling procedures. The similarities of our results with those obtained analyzing blow microbial communities of humpback whales [10], the findings of bacterial Genera reported also in other cetaceans’ respiratory and skin microbiome [27,28,29,30, 32], the peculiarities of cetaceans’ respiratory microbiota and the absence of the same bacterial species in the seawater sample confirm the success of our sampling efforts by using the above described tool.

In conclusion, our research confirms the possibility of using UAVs to collect exhaled air in small cetaceans, despite the significant logistical and technical challenges. An adequate tool suitable for sampling exhaled air from dolphins, like the one presented in this paper, could overcome difficulties in sampling exhaled air causing by animals’ swimming speed, unpredictable swimming pattern, respiratory rate, and social structure. Although this approach should be improved, it represents an important advancement in the health assessment of wild dolphins. Future studies could be aimed to implement this sampling methodology for pathogen screening and hormone quantification in exhaled air. In addition, the possibility of mounting high-resolution cameras on the UAVs would not only provide the ability to integrate photogrammetry and thermal imaging into the dataset, but would also allow us to link the results to specific photo-identified individuals.

Exhaled breath sampling with UAVs provides an additional, noninvasive approach to assess and monitor the health status of wild dolphins. Further investigations could relate respiratory microbiome with viral populations, hormonal and cytokine biomarkers exhaled with the air, and for any evidences of respiratory disease. For instance, performing proper investigations comparing microbial communities and pathological changes during postmortem investigations of dolphins maintained *ex-situ* for a long period of time, and on freshly stranded individuals, could enhance the screening and health monitoring of wild cetacean populations [13, 35].
