# Correction: The use of Unmanned Aerial Vehicles (UAVs) to sample the blow microbiome of small cetaceans

**DOI:** 10.1371/journal.pone.0246177

**Published:** 2021-01-22

**Authors:** Cinzia Centelleghe, Lisa Carraro, Joan Gonzalvo, Massimiliano Rosso, Erika Esposti, Claudia Gili, Marco Bonato, Davide Pedrotti, Barbara Cardazzo, Michele Povinelli, Sandro Mazzariol

There is an error in reference 10. The correct reference is: Pirotta V, Smith A, Ostrowski M, Russell D, Jonsen ID, Grech A and Harcourt R (2017) An Economical Custom-Built Drone for Assessing Whale Health. Front. Mar. Sci. 4:425. doi: 10.3389/fmars.2017.00425.
